# Potential missed opportunities for organ donation: incidence and risk factors for non-referral among critically ill patients

**DOI:** 10.3389/frtra.2026.1924053

**Published:** 2026-09-07

**Authors:** Leda Nobile, Chiara Faso, Gaia Furlan, Filippo Annoni, Vincent Labbé, Mehdi Mezidi, Valerio Lucidi, Thierry Gustot, Jean-Louis Vincent, Fabio Silvio Taccone

**Affiliations:** 1 Department of Intensive Care, Hôpital Universitaire de Bruxelles (HUB), Université Libre de Bruxelles (ULB), Brussels, Belgium; 2 Transplantation Unit, Hôpital Universitaire de Bruxelles (HUB), Université Libre de Bruxelles (ULB), Brussels, Belgium

**Keywords:** brain death, donor pool, end-of-life decision, neurocritical care, organ procurement, organ shortage, potential organ donor, risk factors

## Abstract

**Introduction:**

Many potential organ donors are not referred to the transplant coordination, constituting potential missed referrals.

**Methods:**

In this retrospective analysis, we explored risk factors for potential missed referral among patients suffering from acute brain injury, who were admitted to a 32-bed mixed intensive care unit (ICU) between 2018 and 2023 and died after hypoxic-ischemic encephalopathy, traumatic brain injury, or subarachnoid hemorrhage (SAH). Patients with absolute contraindications for donation were excluded. Each patient was categorized as “actual donor”, “medical refusal”, “personal refusal”, or “potential missed referral”. The primary outcome was the potential missed referral rate.

**Results:**

Of 183 potential donors, 101 (55%) were referred and 82 (45%) were not; on ICU admission, patients in the potential missed referral group were older, had higher severity scores, more comorbidities, and were less likely to have a diagnosis of SAH. In a binomial logistic regression analysis, older age and chronic obstructive pulmonary disease were independent predictors of potential missed referral; donation after brain death and SAH were associated with higher referral rates.

**Conclusions:**

This audit identifies areas for improvement to expand the donor pool, including greater recognition of the donor potential of critically ill patients with extra-neurological complications, even in the presence of older age and comorbidities.

## Introduction

Solid organ transplantation can be the definitive treatment for end-stage organ disease, yet growing demand has widened the gap between organ availability and patients awaiting transplantation (1–4). A systematic approach, i.e., the critical pathway, to the deceased organ donation process was developed in 2011 to facilitate early detection and referral of potential donors (5). A potential deceased organ donor was defined as a patient who had sustained a devastating brain injury but was considered medically suitable for organ donation. Two pathways were discussed that may lead to possible organ donation: donation after circulatory death (DCD) refers to the situation when circulatory and respiratory functions have stopped and resuscitative measures are not to be initiated or continued, or when such functions are expected to cease within a timeframe compatible with organ retrieval; donation after brain death (DBD) refers to the situation when any residual cerebral function has ceased in the absence of confounders and brain death is confirmed using adequate ancillary testing (5).

Although the most common pathologies leading to possible organ donation, including hypoxic-ischemic encephalopathy (HIE) following cardiac arrest, traumatic brain injury (TBI), or subarachnoid hemorrhage (SAH), are well characterized (4), identification of affected patients as potential donors remains suboptimal (6). A donation physician program has been recommended by the government of Alberta in Canada, to help drive awareness of all aspects of the organ donation process, from prognostication through early identification to correct management (7). The importance of having a dedicated professional responsible for every aspect of organ donation was confirmed in a recent Canadian study (8); implementation of a donation physician program was associated with a significant decrease in missed referrals of potential organ donors after 3 years. The same authors (9) reported that age, death in the emergency department or at a regional hospital, and DCD were independent predictors of missed donation opportunities. In Europe, guidelines for organ transplantation (6) also emphasize the need to refer any possible organ donor immediately upon identification and the important role of transplant coordinators early in the donation pathway.

In Belgium, although legislation (10) mandates that every hospital establish a multidisciplinary organ donation team and provide dedicated training for donor identification and management, implementation remains heterogeneous. Healthcare professionals responsible for organ donation frequently combine these responsibilities with routine intensive care activities, and structured certification programs currently exist only for nurses (11), with no equivalent standardized curriculum for physicians. Consequently, barriers to timely donor recognition and referral may persist despite an established legislative framework. Identifying these barriers is essential to optimize referral pathways, develop targeted educational interventions, and reduce missed donation opportunities.

We therefore investigated the prevalence and determinants of missed organ donation opportunities among ICU patients with HIE, TBI, or SAH, three conditions accounting for the majority of deceased organ donors.

## Patients and methods

### Study design

This retrospective analysis of data from the 32-bed mixed Department of Intensive Care at the Hôpital Universitaire de Bruxelles (HUB), Brussels (Belgium) was conducted according to the STROBE statement for cohort studies (12). The local Ethics Committee (Comité d'Éthique Hospitalo-Facultaire Erasme, ULB) approved the study (P2024/299) and waived the need for informed consent because of its retrospective nature and the deceased status of the patients.

### Study population

Patients admitted to the Department of Intensive Care between January 2018 and December 2023 who died after a diagnosis of HIE, TBI, or SAH were identified from three institutional databases (one for each disease) and were considered for inclusion. Our institutional referral policy states that all potential organ donors, regardless their age, expected time to death after withdrawal of life-sustaining treatment and organ dysfunction, not presenting a clear absolute contraindication (see below) must be reported to the transplant coordination (TC).

Among the HIE patients, only patients with a sustained return of spontaneous circulation were considered as potential donors (13), given the absence of an active Maastricht-II (14) donation program (i.e., after unsuccessful resuscitation) in our center. Patients with absolute organ donor contraindications according to the Guide to the Quality and Safety of Organs For Transplantation (15), such as active malignancy or severe disseminated infection, were excluded. Four additional contraindications were applied in accordance with our institutional protocols: hepatitis C virus (HCV) infection (prior to 2022); human immunodeficiency virus (HIV) infection; known intravenous drug abuse; and a positive assay for severe acute respiratory syndrome coronavirus-2 (SARS-CoV-2; between March 2020 and January 2022). In all three groups (HIE, TBI, and SAH), patients with shock or multiorgan failure (MOF, defined as simultaneous failure of at least two organs, i.e., organ-specific Sequential Organ Failure Assessment, SOFA score of 3–4) were considered potential organ donors only when these conditions followed a decision to withdraw or withhold life-sustaining treatment. Patients in whom shock or MOF developed despite maximal medical therapy were excluded, as organ viability could not be assured, justifying a non-referral strategy.

### Classification of the potential organ donors

We cross-matched our database with the potential organ donor database [a mandatory national registry (16) to which all hospitals involved in organ donation must report the number of potential donors, actual donors, organs transplanted, and reasons for not proceeding with organ retrieval] to ensure all eligible cases were identified. This mandatory database is maintained by a designated individual, who is informed of all organ donation discussions (whether with the family or the transplant coordinator), ensuring that every potential donor is recorded. We characterized the potential organ donors into a “referred” group, comprising all cases referred to the transplant coordination team, including “actual donors” (i.e., organ donation performed), “personal refusal” (i.e., donation refused by family), and “medical refusal” (i.e., donation refused for medical reasons by the transplant coordination team). Other potential organ donors were included in the “potential missed-referral” group. We defined a potential missed organ donation attributable to the ICU team as the failure to refer a potential donor to the TC in the absence of an absolute contraindication or of major, unavoidable hemodynamic instability. Other elements, such as the TC own assessment of the donor suitability or the expected time to death after withdrawal of life-sustaining therapies, apply only once a donor has been identified and referred; they therefore fall outside the ICU team's referral decision and were not considered in defining a potential missed referral.

### Data collection

Patient demographics, comorbidities, and the SOFA score (17) and Simplified Acute Physiology Score (SAPS) III (18) at ICU admission were collected. Data collection on admission was chosen as the reference time point for two reasons: first, SAPS III is designed and validated for computation from variables collected within one hour of ICU admission, and its calculation at a later time point would fall outside its intended use. Second, our objective was not to describe the patient's condition at the moment of possible donor recognition, but to examine whether the severity of illness apparent at the outset of the ICU stay was associated with the subsequent likelihood of a potential donor being referred to the transplant coordinator.

The time and cause of death, presence of an infection, data on respiratory, hepatic, and renal function, and chest x-ray findings were recorded on the day of death. Infection was not adjudicated retrospectively against standardized diagnostic criteria; a patient was therefore considered infected if a diagnosis of infection was documented by the treating physician in the medical chart on the day of death. This time point was chosen deliberately: our aim was to capture the clinician's assessment as it stood at the time of the referral decision, since it is the perceived, rather than microbiologically confirmed, infectious status that could plausibly have influenced the decision to refer or not refer a potential donor.

The time of death was dichotomized into “day shift” (i.e., occurring between 9 am and 8 pm) and “night shift” (i.e., occurring between 8 pm and 9 am), and separately into “early” [when the length of ICU stay (LOS) was ≤ 2 days] or “late” (when the LOS was > 2 days). The cause of death was classified as neurological, dichotomized into DBD (19) or “severe neurological impairment” (i.e., leading to withdrawal or withholding of life-sustaining therapies), or non-neurological (shock, respiratory failure, mesenteric ischemia, or MOF). Shock was defined as hypotension with clinical and biological signs of tissue hypoperfusion (i.e., mottled skin, prolonged capillary refill time, oliguria, lactate >2 mmol/L) (20). Respiratory failure included acute respiratory distress syndrome (ARDS) defined according to the Berlin criteria (21), as well as non-ARDS cases.

### Study outcomes

The primary outcome was the rate of potential missed referrals. Secondary outcomes included rates of medical refusals, personal refusals for the whole population and according to DBD and DCD categories. The identification and referral rate (IDR), calculated as the proportion of identified and referred potential organ donors among all potential donors, was also assessed (22).

### Statistical analysis

No sample calculation was performed due to the retrospective nature of this study. Analyses were performed using IBM SPSS Statistics 21 for Windows and GraphPad Prism software, V10.3.1. Descriptive statistics were computed for all study variables, and normal distribution was assessed using the Kolmogorov–Smirnov test. Data are presented as count (percentage) or median [25th–75th percentiles]. Differences between groups were assessed using chi-square or Fisher's exact test for categorical variables, and a Mann Whitney U-test for continuous variables. For the IDR, 95% confidence intervals were calculated using the Wilson method. Potential predictors associated with the primary outcome in univariable analyses were entered into the multivariable model, except for those resulting in complete separation. Variables with pairwise correlations greater than 0.5 were excluded to limit multicollinearity. A multivariable logistic regression model was then built using backward elimination according to Wald criteria, retaining a final model with a non-significant Hosmer–Lemeshow goodness-of-fit test. Model parameters were reported as odds ratios (ORs) with 95% confidence intervals (CIs) and *p*-values. To increase the reliability of point estimates, model parameters were subsequently estimated using nonparametric bootstrap resampling with 1,000 replications. Age was analyzed in 10-year intervals to evaluate the change in risk of being misreferred associated with each decade increase. No correction for multiple testing was applied. A *p*-value < 0.05 was considered statistically significant.

## Results

### Study population

**Figure 1:**
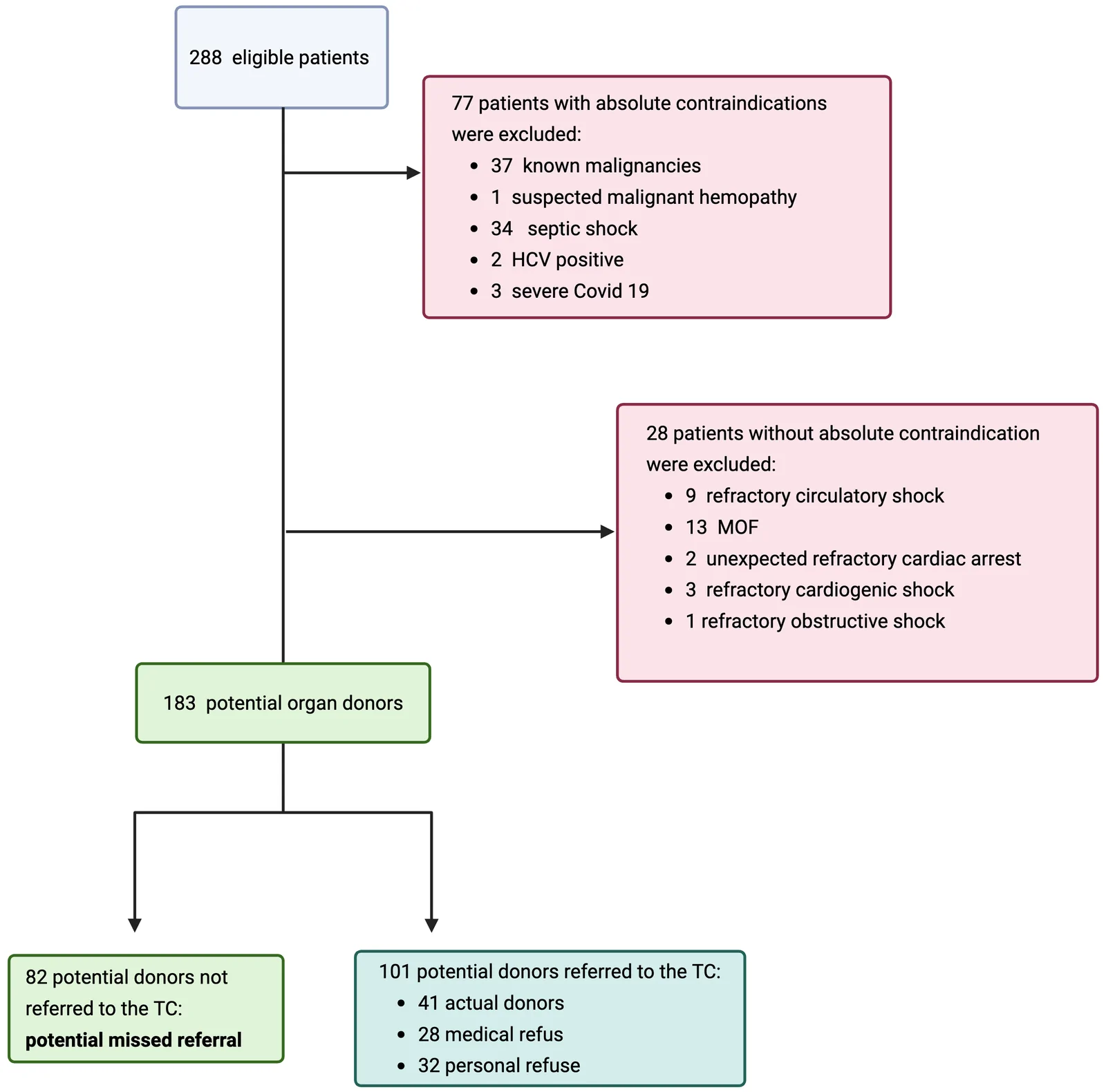
Flowchart of potential organ donor screening.

**Figure 2:**
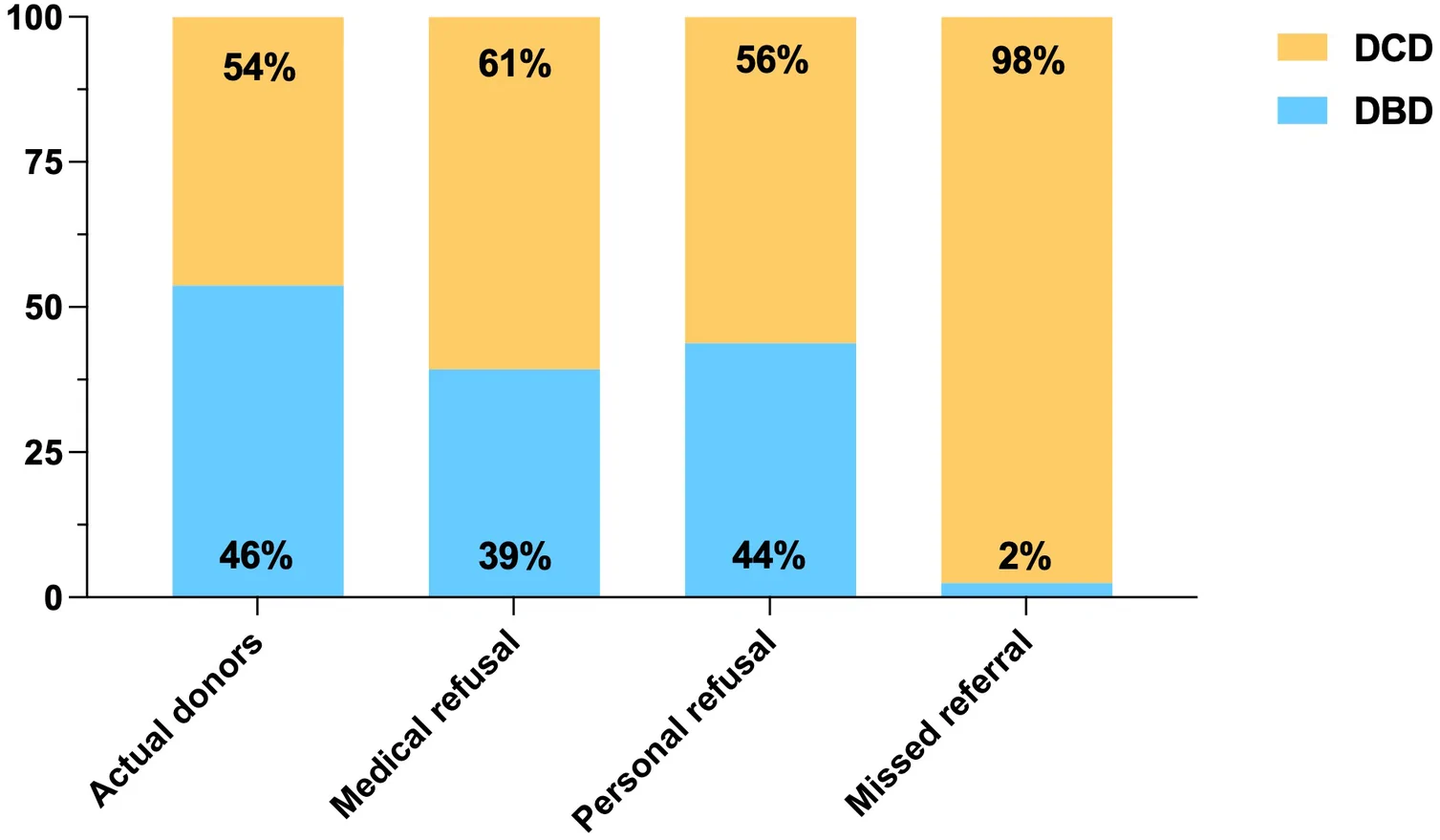
Distribution of actual donors, medical refusal, personal refusal, and potential missed referral according to the type of donor.

During the study period, 288 patients admitted for HIE (*n* = 174), TBI (*n* = 73), or SAH (*n* = 41) died during the ICU stay: 77 of these patients had an absolute contraindication to organ donation and 28 were excluded for circulatory failure or MOF despite maximal appropriate medical treatment, leading to a cohort of 183 potential donors (Figure 1). The median age was 66 [48–76] years, and 60% of patients were men (Table 1). One hundred and one (55%) potential organ donors were referred to the transplant coordination team (referral group), including 41 (41%) actual donors, 28 (28%) medical refusals, and 32 (32%) personal refusals. The IDR in the whole study cohort was thus 0.55 (95% CI 0.48–0.62). In the subgroup of DBD potential donors (*n* = 49), there were 22 (45%) actual donors, 11 (22%) medical refusals, 14 (29%) personal refusals, and 2 (4%) potential missed referrals; the IDR was 0.96 (CI 95% 0.86–0.99). In the DCD potential donors (*n* = 134), there were 19 (14%) actual donors, 17 (13%) medical refusals, 18 (13%) personal refusals, and 80 (60%) potential missed referrals; the IDR was 0.4 (CI 95% 0.32–0.49). A significant difference in potential missed referrals was therefore observed between DCD and DBD categories (Figure 2).

**Table 1:** Baseline patient characteristics according to whether or not they were referred to the transplantation coordination team.

Variable	Non-referred (*n* = 82)	Referred (*n* = 101)	*P* value
Age, years	73 [66–80]	57 [43–67]	<0.001
Men, *n* (%)	49 (60)	61 (60)	>0.99
Past medical history			
Alcohol use, *n* (%)	21 (26)	23 (23)	0.73
Cigarette smoking, *n* (%)	12 (15)	28 (28)	0.047
Diabetes mellitus, *n* (%)	18 (22)	12 (12)	0.07
Arterial hypertension, *n* (%)	49 (60)	37 (37)	0.003
Cardiac disease, *n* (%)	40 (49)	32 (32)	0.02
Chronic kidney failure, *n* (%)	13 (16)	8 (8)	0.10
Cirrhosis, *n* (%)	7 (8)	-	0.003
Immunosuppression therapy, *n* (%)	5 (6)	4 (4)	0.52
COPD, *n* (%)	12 (15)	3 (3)	0.006
Admission diagnosis			
Traumatic brain injury, *n* (%)	29 (35)	25 (25)	0.14
Subarachnoid hemorrhage, *n* (%)	7 (8)	21 (21)	0.024
Hypoxic-ischemic encephalopathy, *n* (%)	46 (56)	55 (54)	0.88
Severity scores on admission			
SOFA score^a^	10 [6–12]	9 [6–10]	0.18
SAPS III score^b^	80 [70–86]	74 [69–80]	0.013

Data are reported as medians [25th–75th percentile] for continuous variables and numbers (percentages) for categorical ones. No correction for multiple testing was applied.

COPD, chronic obstructive pulmonary disease; SOFA, sequential organ failure assessment; SAPS III, simplified acute physiology score III.

a SOFA score ranges from 0 to 24.

b SAPS III score ranges from 0 (lowest) to 242 (highest).

Patients in the potential missed referral group (*n* = 82, 45%) were older, had a higher SAPS III score on admission, and were more likely to have comorbid hypertension, cardiac disease, COPD, or cirrhosis than patients in the referred group; these patients less often had a diagnosis of SAH (Table 1). Notably, all patients with liver cirrhosis were in the potential missed referral group. The median ICU LOS was higher in the potential missed referral group. On the day of death (Table 2), potential missed referral patients had higher serum urea levels, lower serum aspartate aminotransferase (AST) levels, and lower daily urine output than those in the referred group. All 13 patients who died of a non-neurological cause were in the potential missed referral group (15%). These deaths were the result of decisions to limit therapy escalation in patients with refractory cardiac conditions (*n* = 4), terminal respiratory failure (*n* = 6), complications of mesenteric ischemia (*n* = 2), and hemorrhagic shock (*n* = 1) (Table 2**)**.

**Table 2:** Laboratory and clinical findings on the day of death according to whether or not a referral was made to the transplant coordination team.

Laboratory and clinical findings	Non- referred (*n* = 82)	Referred (*n* = 101)	*P* value
ICU LOS, days	4 [2–10]	3[1–5]	0.03
Night shift, *n* (%)	24 (29)	32 (32)	0.75
Weekend, *n* (%)	19 (23)	22 (22)	0.86
COVID-19 period, *n* (%)	11 (13)	7 (7)	0.21
Laboratory findings on the day of death			
Alanine aminotransferase, IU/L	39 [22–82]	51 [26–92]	0.15
Aspartate aminotransferase, IU/L	46 [ 30–73]	62 [40–128]	0.013
Serum creatinine, mg/dL	0.99 [0.6–1.5]	0.8 [0.6–1.2]	0.054
Urea, mg/dL	50 [33–80]	31 [21–48]	<0.001
Daily urine output, mL	1,050 [310–1,904]	2,000 [845–2,965]	0.001
PaO_2_/FiO_2_ ratio	236 [172–307]^a^	265 [180–361]^b^	0.07
Infections on the day of death			
Infected patients	34 (41)	34 (34)	0.28
Lung, *n* (%)	27 (33)	31 (31)	0.75
Causes of death			
Neurological, *n* (%)	69 (84)	101 (100)	<0.001
Brain death, *n* (%)	2 (2)	47 (46)	<0.001
Severe neurological impairment, *n* (%)	67 (82)	54 (53)	<0.01

Data are reported as medians [25th–75th percentile] for continuous variables and numbers (percentages) for categorical ones. No correction for multiple testing was applied.

ICU LOS, intensive care unit length of stay; MOF, multiorgan failure; COVID-19, Coronavirus disease 2019.

a missing values = 18.

b missing values =5.

### Factors associated with potential missed referral

In the univariate analysis, older age, higher SAPS III score on admission, longer ICU LOS, comorbid arterial hypertension, cardiac disease, liver cirrhosis, or chronic obstructive pulmonary disease (COPD), elevated urea, and lower AST or urine output on the day of the death were associated with potential missed referral. SAH as a cause of ICU admission and DBD were associated with a reduced risk of potential missed referral.

In the binomial logistic regression, the final model was the most parsimonious while maintaining adequate goodness of fit (Hosmer–Lemeshow test *χ*² = 10.8, df = 8, *p* = 0.21). The model included DBD, ICU LOS, COPD, type of underlying brain injury, urine output on the day of death, and age. Factors associated with a lower risk of potential missed referral were DBD (OR 0.04, 95%CI 0.01–0.2) and SAH (OR 0.20, 95%CI 0.04–0.7). A history of COPD (OR 5.5, 95% CI 1.15–26.5) and older age (OR 1.6, CI 95% 1.2–2.1) were independently associated with a higher probability of potential missed referral (Table 3). After performing the bootstrap (Table 3), there was a marked widening of the 95% CI for COPD (2.1–1.2 × 10^9^ vs. 1.15–26.5 in the binomial logistic regression).

**Table 3:** Binomial logistic regression and bootstrap of factors associated with potential missed referral.

	Binomial logistic regression		Bootstrap of binomial logistic regression	
Variable	OR (95% CI)	*p* value	OR (95% CI)	*p* value
Age (per 10 y)	1.6 (1.2–2.1)	<0.001	1.6 (1.2–2.7)	0.004
SAH^a^	0.2 (0.04–0.7)	0.02	0.16 (0.02–0.8)	0.01
HIE^a^	0.6 (0.2–1.4)	0.24	0.6 (0.18–1.8)	0.32
COPD	5.54 (1.15–26.5)	0.03	5.54 (2.1–1.2 × 109)	<0.001
LOS (per day)	1.04 (0.99–1.1)	0.08	1.04 (0.99–1.1)	0.054
Urine output (last day per L)	1.2 (0.97–1.5)	0.09	1.2 (0.89–1.7)	0.12
Brain death	0.04 (0.01–0.2)	<0.001	0.04 (<0.01–0.14)	<0.001

OR, odd ratio; CI, confidence interval; TBI, traumatic brain injury; HIE, hypoxic ischemic encephalopathy; SAH, subarachnoid hemorrhage; COPD, chronic obstructive pulmonary disease; LOS, length of stay.

a compared to TBI.

## Discussion

In this single-center review of patients who died in the ICU over a 5-year period, older age and a history of COPD were independent predictors of potential missed referral for potential organ donors. In contrast, DBD and SAH as a reason for ICU admission were significantly associated with a reduced risk of potential missed referral.

Kramer et al. recently reported that older age, death in the emergency department or at a regional hospital, and DCD were the most relevant predictors of missed donation opportunities (9). Our findings are consistent with these results. However, in the Kramer's study, potential organ donors with circulatory instability and older patients (i.e., > 70 years old for DCDs) were excluded as considered not eligible for organ donation. No age restriction was applied to our analysis, because, in Belgium, the oldest DCD organ donor in 2023 was a 94-year-old who donated both the lungs and the liver. We also decided not to exclude circulatory instability if it was the consequence of deliberate therapeutic limitation, as previously mentioned. Finally, we did not analyze the location of death as all patients with devastating brain injury are only managed by the ICU team in our institution.

A possible explanation for the apparent “blinding effect” of age and comorbidities (such as COPD and cirrhosis) on donor referral is that ICU physicians may apply overly restrictive criteria when assessing donor suitability, leading to the exclusion of older patients and those with comorbid conditions. The expansion of acceptance criteria and the inclusion of non-standard risk donors have been pivotal advances in contemporary transplantation, as shown in Spain, which has achieved one of the highest donor rates globally (23). Central to the Spanish strategy has been the systematic use of expanded criteria donors, with more than 10% of donors aged over 80 years. Another cornerstone of the Spanish model is the distinctive role of the transplant coordinator, typically a critical care physician, who is responsible for donor identification, referral, and management, and for continuous training of ICU staff in donor identification and the donation process. In Belgium, such a strategy is not fully implemented; even if the law (16) requires a local team dedicated to identifying and managing potential organ donors, consisting of at least a medical doctor and a nurse, these healthcare providers are not always specifically assigned to this task. They often must manage, at the same time, both ICU and donation responsibilities. Importantly, although COPD emerged as an independent predictor in the multivariable model, this finding should be interpreted with caution. The extremely wide bootstrap confidence interval indicates considerable instability of the effect estimate and suggests limited precision of the model for this variable. Such imprecision may reflect sparse data, a limited number of events, or model overfitting, and reduces confidence that the observed association represents a robust independent effect.

*Post-mortem* strategies to optimize organ preservation, such as reconditioning using normothermic regional perfusion or hypothermic pulsatile perfusion, have also contributed to enhancing graft viability thus increasing the potential donor pool (24, 25). Among our cohort, we performed well in detecting DBD potential donors with a rate of missed referrals lower than expected based on previously published data from Belgium (2). However, our analysis of DCD potential donors showed the opposite trend. One possible explanation for this finding may be provided by the underlying causes of death. By definition, all DBD donors progress to brain death, but the pathway to potential donation begins well before this terminal event.

When comparing missed referrals with referred cases, we observed that all non-neurological causes of death were in the missed referral group. These non-neurological conditions may have been associated with premature withdrawal or withholding of therapies, leading to MOF or untreated shock. This pattern suggests that ICU physicians readily identify potential donors who are clearly progressing toward brain death. In contrast, when the neurological prognosis is considered very poor but brain death criteria have not yet been met, development of extra-neurological complications appears to prompt early withdrawal of life-sustaining treatment with no attempt to manage those complications. This tendency likely reflects the perception that treating extra-neurological complications in a patient with a devastating neurological injury is futile, a rationale that may be valid when the sole objective is survival of the patient, but becomes more difficult to justify if organ donation is considered a realistic outcome. Importantly, our findings should not be interpreted as suggesting that life-sustaining therapies should be continued primarily to facilitate organ donation (26). Decisions regarding neurological prognostication and withdrawal or withholding of life-sustaining treatment must remain entirely independent of considerations related to organ donation and should be based exclusively on the patient's prognosis, values, and best interests. Rather, our results suggest that potential donor referral may sometimes be overlooked when severe extracerebral complications develop before brain death has occurred. In such circumstances, once an independent end-of-life decision has been reached and organ donation remains a realistic possibility, timely referral to the transplant coordination team allows donor suitability to be assessed by professionals with specific expertise, avoiding premature exclusion of potential donors based solely on the treating team's perception of medical suitability.

According to the Neurocritical Care Society recommendations (24), a waiting period of 72 h after the initial injury is strongly suggested before assessing neurological prognosis. This delay helps prevent hasty decisions and self-fulfilling prophecies leading to early withdrawal of life support, which could inadvertently influence the outcome and affect the possibility of organ donation. In some patients in the present cohort, aggressive treatment may have been considered futile because of a poor prognosis, without considering that ongoing organ support could still facilitate successful organ donation. Interestingly, elective non-therapeutic intensive care admissions to facilitate organ donation are a cornerstone of the Spanish model and may contribute to substantially expanding the donor pool (23).

A diagnosis of SAH was associated with higher referral rates than other diagnoses. Exploratory *post-hoc* analyses revealed longer ICU LOS in patients with SAH, likely due to prolonged management of complications such as vasospasm-induced ischemia. This prolonged ICU management reflects the natural history of the disease, particularly with the occurrence of delayed cerebral ischemia and/or cerebral vasospasm (27), and likely contributes to physician recognition and acceptance of treatment futility. Consequently, it may increase the perceived appropriateness of organ donation proposal, and thus the higher referral rates, compared to the situation with sudden catastrophic injuries, such as TBI (often with extracranial trauma) or HIE [complicated by cardiac or circulatory failure (28)].

In our cohort overall, 22% were actual donors, 15% medical refusals, 17% personal refusals, and 45% potential missed referrals, yielding an IDR of 0.55. To facilitate comparison with published series, we stratified the population into DBD and DCD subgroups. Our findings only partially align with previous reports. In 2016 in Belgium, Hoste et al. observed a lower proportion of actual donors, a higher proportion of missed referrals, and lower rates of both medical and personal refusals within the DBD population (2). The IDR in our cohort was markedly lower than that reported by high-performing Canadian organ donation organizations (0.87) (22), but comparable to less-well performing Canadian organizations (0.55), and to that of Belgium (0.47) during the same study period (29). Within the European context, our IDR was lower than that of the UK (0.86 between 2019 and 2024) (30); the IDR was 0.95 for DBD donors and 0.33 for DCD donors. This discrepancy with our findings may be explained by examining the ratio of referred potential donors to actual donors. In the UK, this ratio is very low (0.23), whereas in Belgium it is much higher (0.89), likely reflecting a more permissive referral policy in the UK, where donation physicians are expected to report all potential donors, even when initial suitability for donation appears limited.

This study has notable strengths. Although recent large population-based studies have already identified older age and the DCD pathway as important determinants of missed donation opportunities, our findings extend these observations to a European tertiary-care transplantation center with different organizational practices and referral policies. More importantly, our analysis focuses specifically on the referral process, the earliest and most modifiable step of the donation pathway, and identifies practical targets for quality improvement, including systematic referral of patients with advanced age, significant comorbidities, and extracerebral complications, particularly within the DCD pathway. Furthermore, the relatively large sample size provided a substantial cohort for classification and analysis.

However, the study also has several limitations. First, it was a single-center retrospective analysis restricted to patients with HIE, TBI, and SAH. This selection ensured data reliability, as these pathologies are systematically captured in prospective registries at our institution. Second, and most importantly, patients retrospectively classified as missed referrals were never evaluated by the transplant coordination team or transplant surgeons. Consequently, organ suitability was not formally assessed, precluding any determination of how many of these patients would ultimately have proceeded to organ donation. Our estimates should therefore be interpreted as the maximum theoretical number of missed donation opportunities rather than the number of potentially transplantable donors. Indeed, a proportion of these patients would likely have been excluded following specialist assessment because of previously unidentified medical contraindications, while additional attrition would inevitably have resulted from family refusal, hemodynamic instability, unsuccessful donor management, or logistical constraints. Accordingly, the present study quantifies the potential for improving donor identification rather than the achievable expansion of the donor pool. Nevertheless, among patients who ultimately died from non-neurological causes, earlier recognition of donation potential might have influenced goals of care, allowing continuation of organ-supportive therapies and thereby preserving the opportunity for organ donation in selected cases. Third, the study period included the COVID-19 pandemic, which may have influenced patient characteristics and causes of death. However, when comparing rates of missed referrals across pre-pandemic, pandemic, and post-pandemic phases, no significant differences were observed (data not shown). Fourth, as a transplantation center managing all solid organ programs, our institution may not represent non-transplant ICUs, in which severe SAH or TBI cases are less frequently managed or referred secondarily to specialized centers. Finally, this study cannot assess human factors that prevent ICU physicians from comfortably integrating organ donation into high-quality end-of-life care. Finally, the retrospective design precluded direct assessment of the cognitive, organizational, and psychosocial factors influencing donor identification and referral. Decisions to initiate discussions regarding organ donation frequently occur under emotionally demanding circumstances and require physicians to transition from a curative to an end-of-life care paradigm. This shift may be particularly challenging when neurological deterioration is progressive rather than sudden, or when prognostic uncertainty persists. Reluctance to approach grieving relatives, concerns that discussing donation may be perceived as inappropriate or intrusive, and limited confidence in conducting end-of-life and donation conversations have all been identified as potential barriers to timely referral. Although these factors could not be formally evaluated in the present study, they likely contribute to the gap between theoretical donor eligibility and actual referral. Structured educational programs specifically addressing communication with families, end-of-life decision-making, and organ donation, such as the European Donor Hospital Education Program (EDHEP) (31), have been available for more than two decades and are widely implemented across Europe (32). However, participation among physicians remains limited in many institutions. Importantly, controlled evaluations of EDHEP suggest that improvements in communication skills may diminish over time in the absence of continued reinforcement, supporting the need for longitudinal, simulation-based, and institutionally embedded educational strategies rather than isolated educational interventions (33). Such programs may improve not only communication with families but also earlier recognition and referral of potential organ donors.

The key message from this study is that patients who were most frequently missed as potential organ donors were older, more likely to have comorbidities, and did not progress to brain death. While these results may not seem surprising, our study provides a valuable audit of current practice. Identification of patients with TBI and HIE as being at higher risk of being missed compared to those with SAH highlights an important area for potential improvement. To expand the donor pool, special attention should be directed toward critically ill patients with extra-neurological complications, even when advanced age and comorbidities are present. By focusing on these profiles and by strengthening awareness among ICU physicians of the potential for donation despite perceived limitations or unsuitability, missed opportunities for referral may be reduced and transplant organ availability ultimately increased. Routine involvement of transplant coordinators may further facilitate this process. In the future, establishment of a larger registry would enable meaningful benchmarking across institutions, help identify local differences, foster better understanding of referral patterns, and provide the basis for guidance to harmonize and improve potential donor recognition.

## Data Availability

The raw data supporting the conclusions of this article will be made available by the authors, without undue reservation.
